# Supplementary material for: The Relative Densities of Cytoplasm and Nuclear Compartments Are Robust against Strong Perturbation
Source: Biophys J. 2020 Oct 20;119(10):1946–57. doi: 10.1016/j.bpj.2020.08.044 (PMC7732746; doi:10.1016/j.bpj.2020.08.044)
Supplement: Document S1. Figs. S1–S9 [file mmc1.pdf]

Biophysical Journal, Volume *119*

Supplemental Information

The Relative Densities of Cytoplasm and Nuclear Compartments Are Robust against Strong Perturbation

Kyoohyun Kim and Jochen Guck

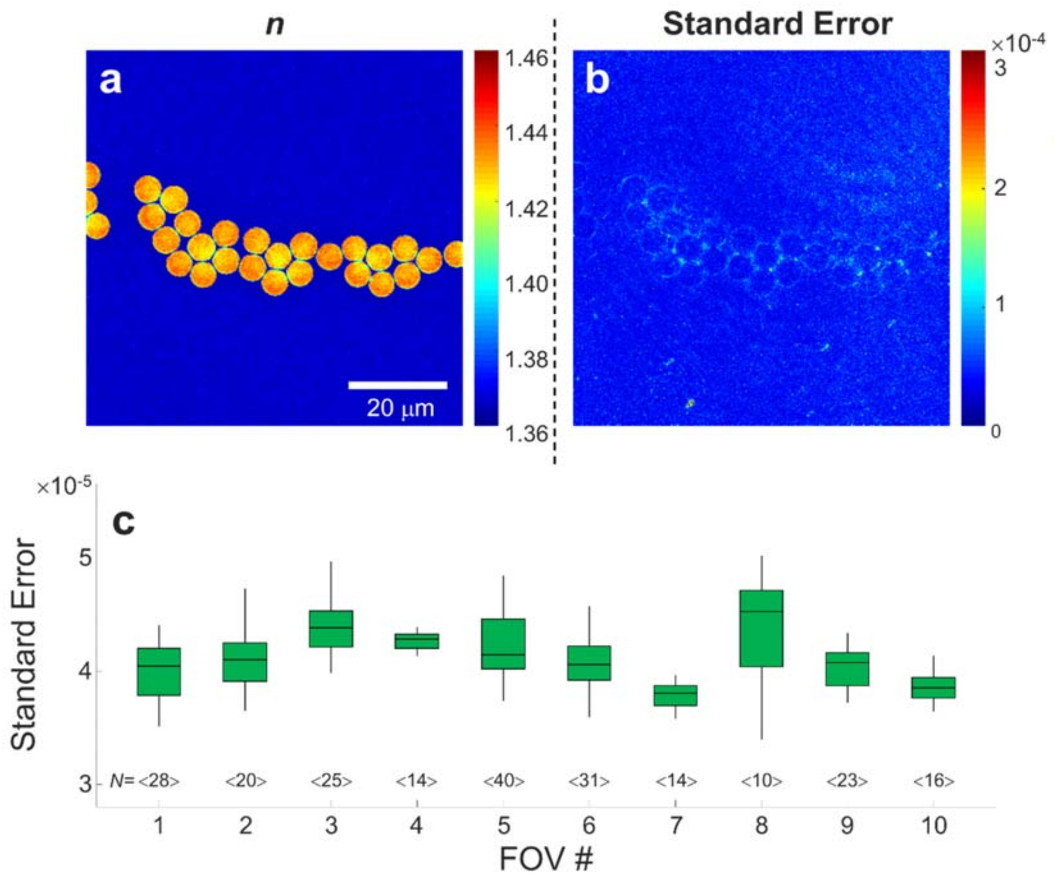

Supplementary Figure 1. Precision of ODT measurements. The cross-sectional slice of (a) an RI tomogram of silica beads immersed in 0.7 M sucrose solution and (b) the standard error of a time series of 10 RI tomograms of the same silica beads. (c) The averaged standard error of a time series of 10 RI tomograms within individual silica bead in each field-of-view (FOV). The number of silica beads in an individual FOV is indicated as *N*. The averaged standard error of RI is calculated as 4.15×10^{-5}.

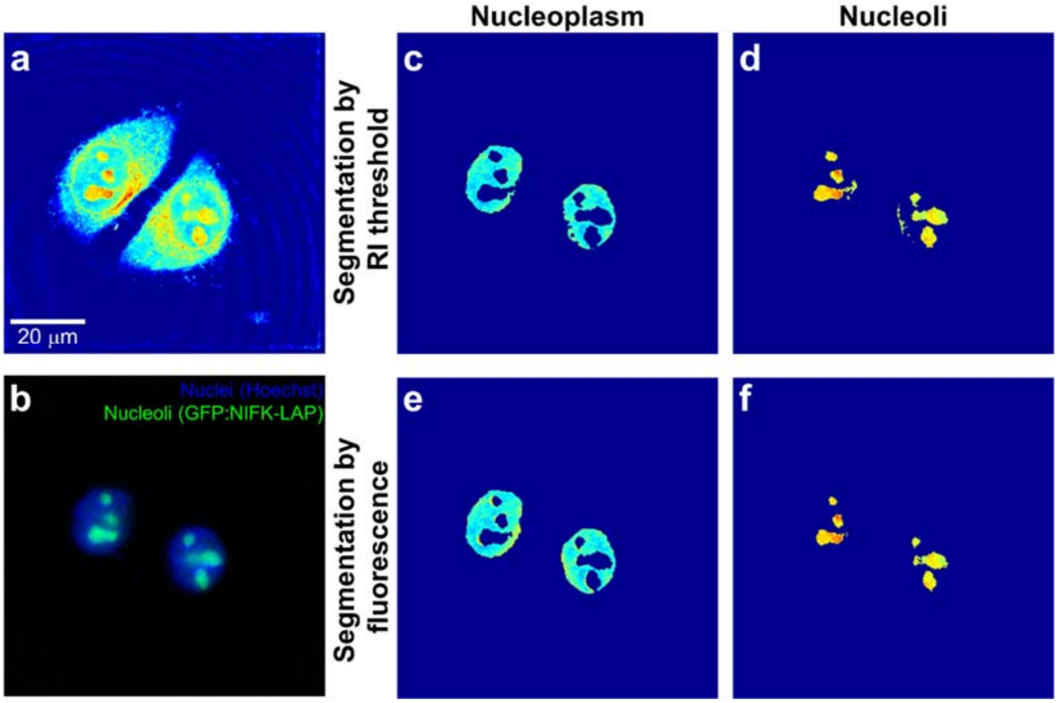

Supplementary Figure 2. Confirmation of the segmentation of nucleoli based on the RI threshold. (a-b) The measured (a) RI tomogram and (b) epi-fluorescence image of HeLa cells which nuclei and nucleoli are stained with Hoechst and GFP, respectively. (c-d) The cross-sectional slices of the RI tomogram of (c) nucleoplasm and (d) nucleoli segmented by the present method based on the RI threshold. (e-f) The cross-sectional slices of the RI tomogram of (e) nucleoplasm and (f) nucleoli segmented by the fluorescence intensity.

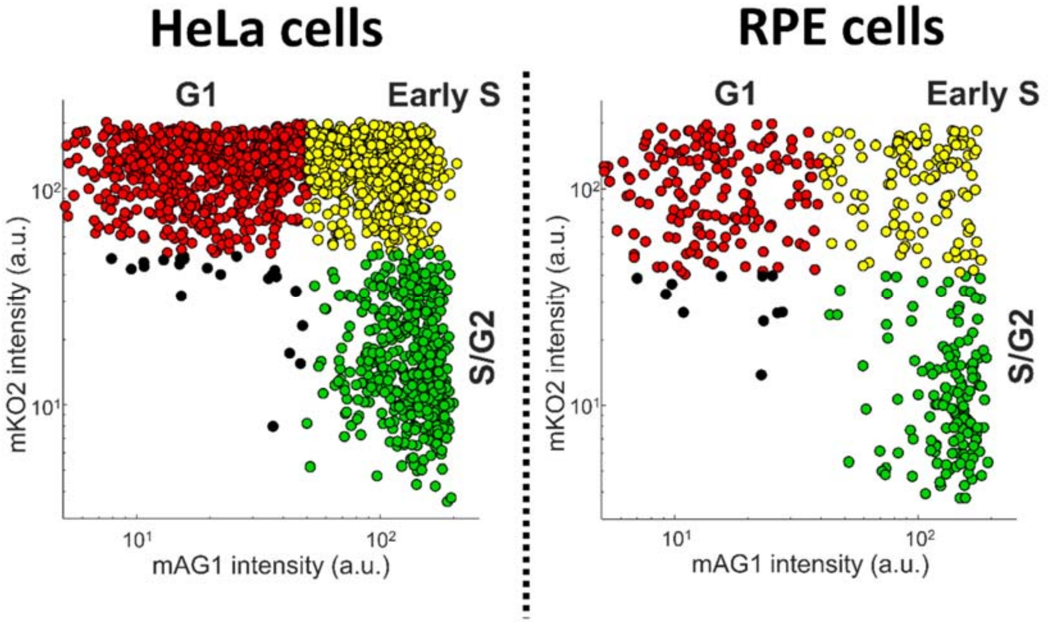

Supplementary Figure 3. Determination of cell cycle phases of individual cells by FUCCI cell-cycle marker. The two-channel epi-fluorescence microscopy measured the intensity of fluorescence signals in FUCCI-stained nucleus in cells (mAG1 and mKO2), and the cell cycle phase of cells was determined in the two-color scatter plot as G1 (high mKO2 signals), early S (high mKO2 and mAG1 signals), and S/G2 (high mAG1 signals) phases. The black dots are determined as either cells in mitosis or cells not expressing fluorescence signals, and discarded in statistical analysis.

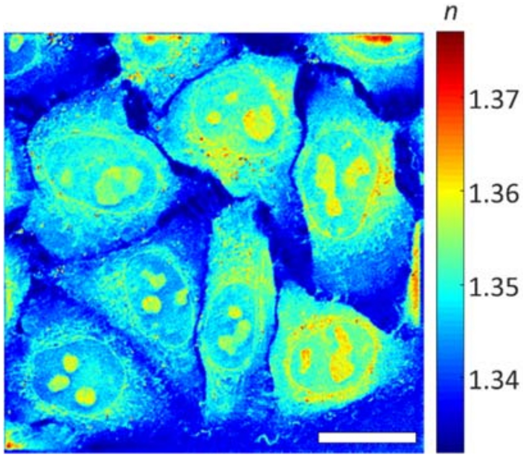

Supplementary Figure 4. Refractive index (RI) and mass density distributions in non-labeled HeLa cells. The maximum projection of an RI tomogram of non-labeled HeLa cells in the direction proportional to the substrate. The scale bar is 20 μm.

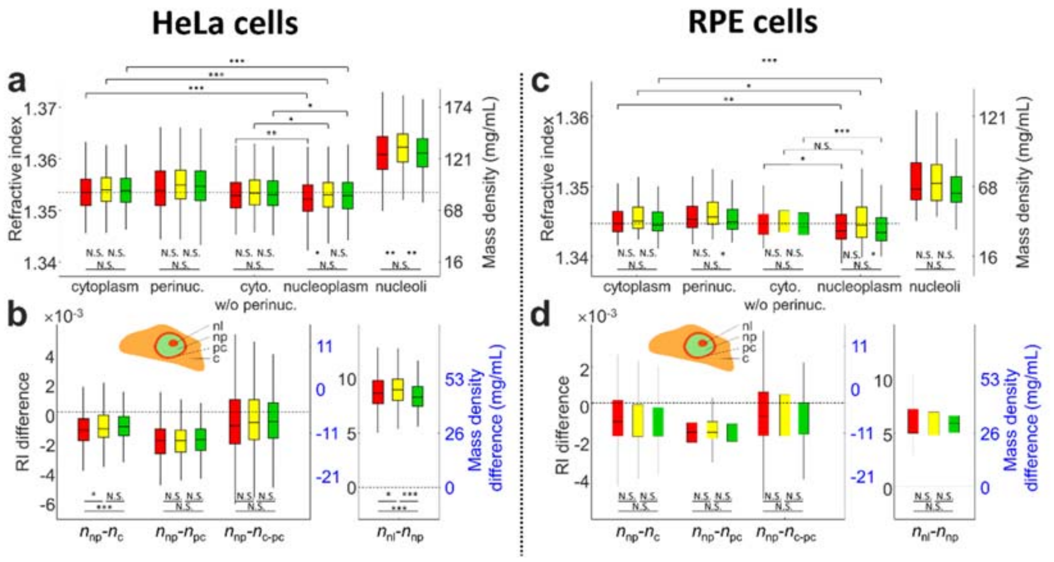

Supplementary Figure 5. Refractive index (RI) and mass density distributions in HeLa-FUCCI (a-b) and RPE-FUCCI (c-d) cells as function of the cell cycle. (a, c) The mean RI values of cytoplasm, perinuclear cytoplasm, cytoplasm excluding perinuclear region, nucleoplasm, and nucleoli. The dashed line indicates the mean RI value of cytoplasm at the G1 phase in the cell cycle. (b, d) The difference of the mean RI values between nucleoplasm and cytoplasm (n_{np}-n_{c}), nucleoplasm and perinuclear cytoplasm (n_{np}-n_{pc}), nucleoplasm and cytoplasm excluding perinuclear region (n_{np}-$n_{c\text{-}pc}$), and nucleoli and nucleoplasm (n_{nl}-n_{np}). The dashed lines indicate the equal mass density of the compartments. The numbers of cells measured are N = 557, 505, and 483 for HeLa cells, and N = 122, 92, and 128 for RPE cells in the G1, early S, and S/G2 phases, respectively.

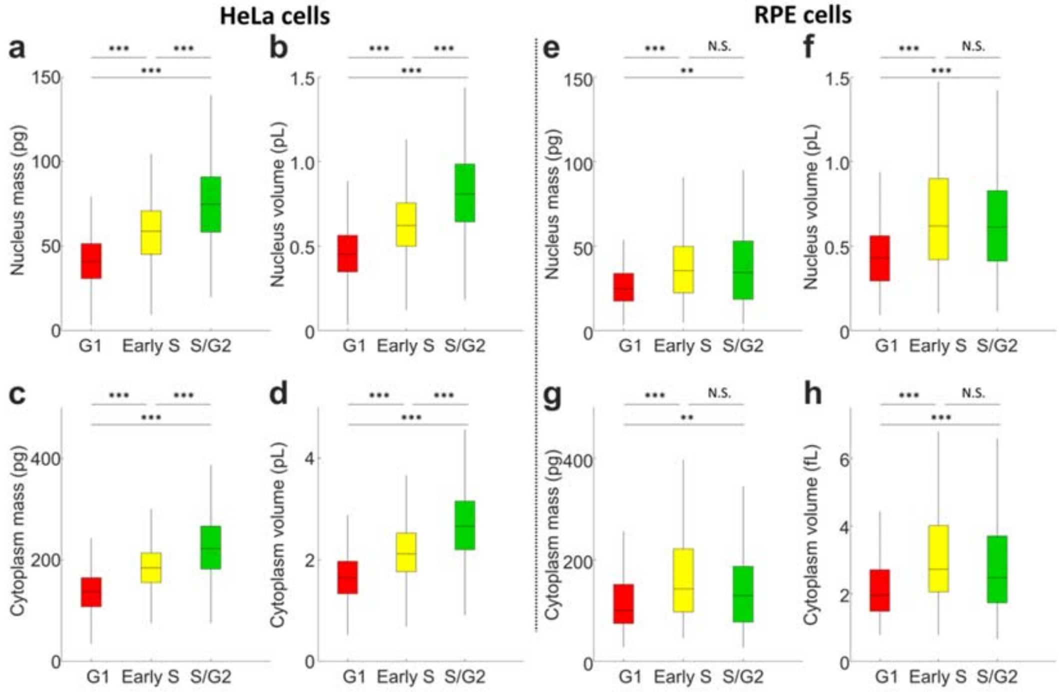

Supplementary Figure 6. Mass and volume distribution of HeLa-FUCCI (a-d) and RPE-FUCCI (e-h) cells as function of the cell cycle. (a, e) The mass and (b, f) volume of nucleus, and (c, g) the mass and (d, h) volume of cytoplasm in HeLa-FUCCI and RPE-FUCCI cells, respectively. The numbers of cells measured are N = 557, 505, and 483 for HeLa cells, and N = 122, 92, and 128 for RPE cells in the G1, early S, and S/G2 phases, respectively.

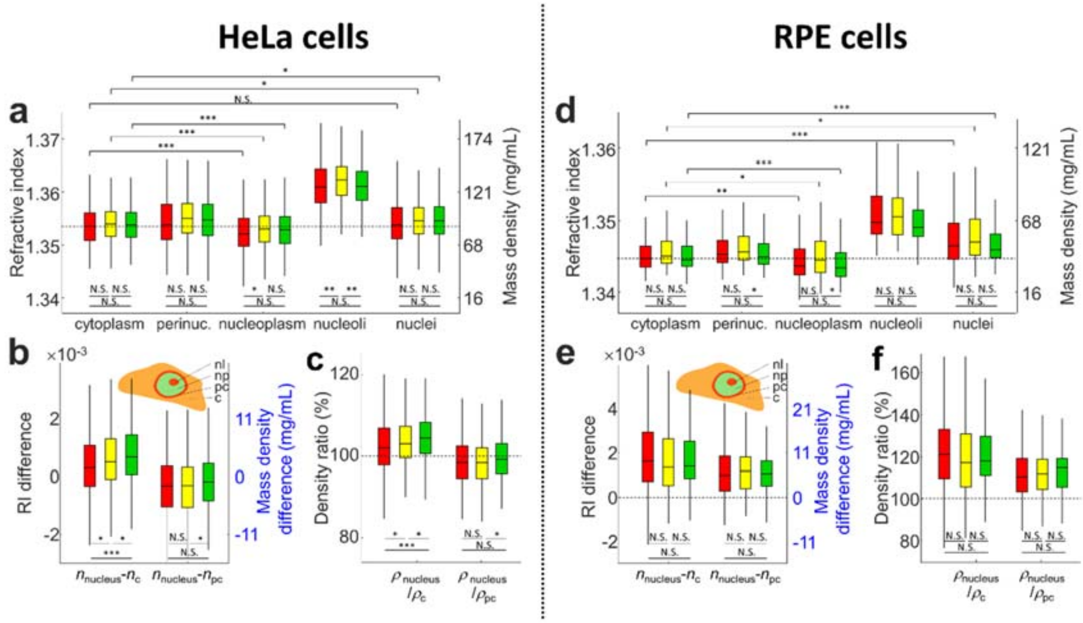

Supplementary Figure 7. Refractive index (RI) and mass density distributions in HeLa-FUCCI (*a*-*c*) and RPE-FUCCI (*d*-*f*) cells as function of the cell cycle. (*a*, *c*) The mean RI values of cytoplasm, perinuclear cytoplasm, nucleoplasm, nucleoli, and nuclei including nucleoplasm and nucleoli. The dashed line indicates the mean RI value of cytoplasm at the G1 phase in the cell cycle. (*b*, *d*) The difference of the mean RI values between nucleus and cytoplasm (n_{np}-n_c) and nucleus and perinuclear cytoplasm (n_{np}-n_{pc}). The dashed lines indicate the equal mass density of the compartments. (*c, f*) The ratio of the mass density between nucleus and cytoplasm ($\rho_{nucleus}/\rho_c$) and nucleus and perinuclear cytoplasm ($\rho_{nucleus}/\rho_{pc}$). The dashed lines indicate equal mass density between compartments. The numbers of cells measured are N = 557, 505, and 483 for HeLa cells, and N = 122, 92, and 128 for RPE cells in the G1, early S, and S/G2 phases, respectively.

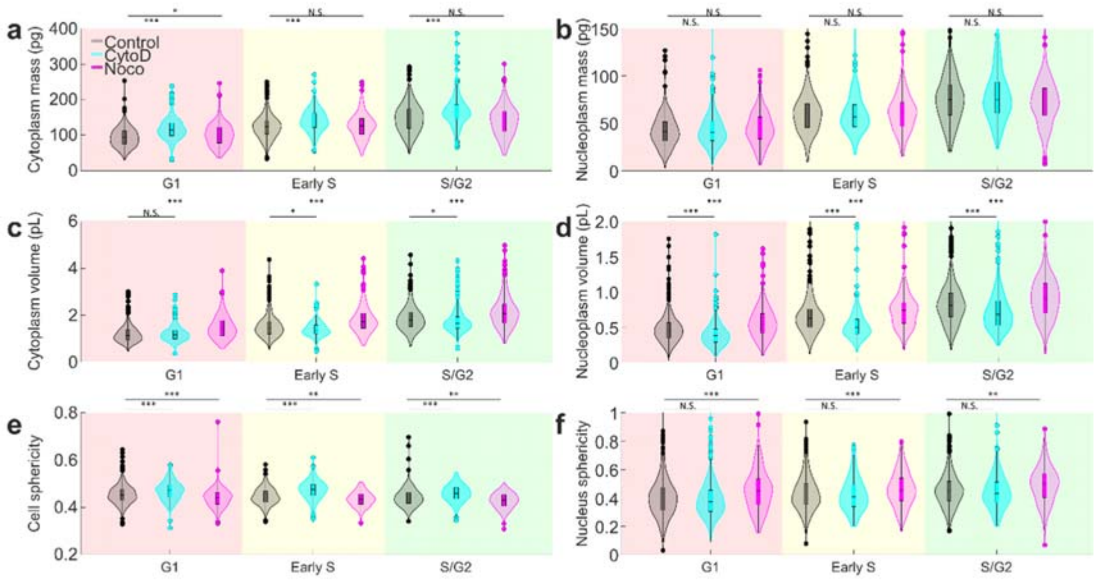

Supplementary Figure 8. Changes in mass, volume, and sphericity of HeLa-FUCCI cells under cytoskeletal perturbation. (a-d) The mass and volume of nucleus and cytoplasm in HeLa-FUCCI cells in control (gray), cytoD (cyan) and noco (magenta) treatment at various cell cycle phases. (e-f) The sphericity of whole cell (e) and nucleus at various cell cycle phases. The cell cycle phases are indicated as shaded region in red (G1), yellow (Early S) and green (S/G2). The numbers of cells measured are N = 1,565, 973, and 717 for control, cytoD, and nocodazole treatments, respectively.

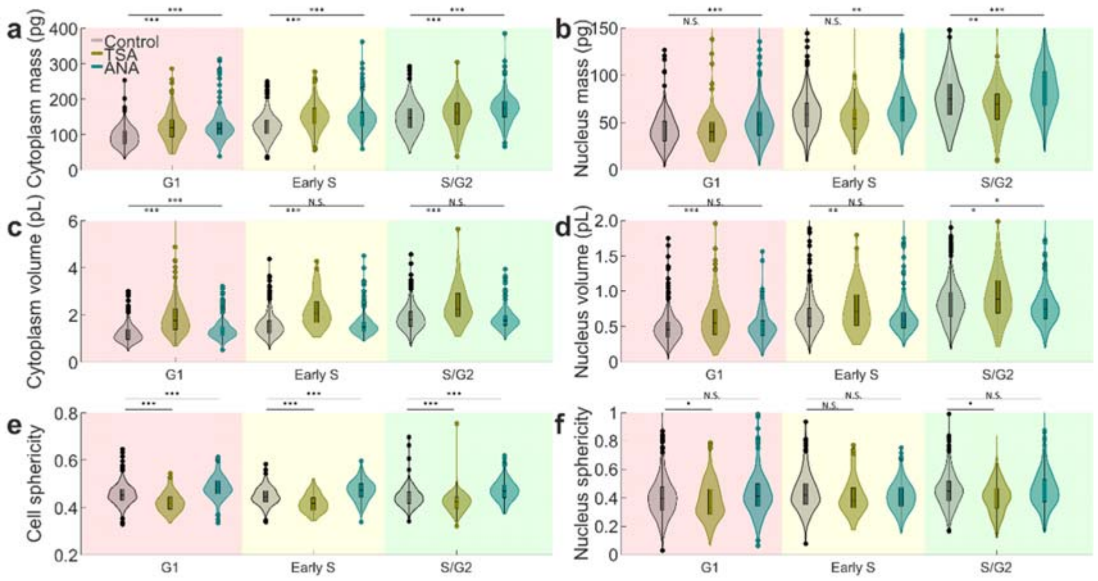

Supplementary Figure 9. Changes in mass, volume, and sphericity of HeLa-FUCCI cells under chromosome condensation. (a-d) The mass and volume of nucleus and cytoplasm in HeLa-FUCCI cells in control (gray), TSA (dark yellow) and ANA (dark green) treatment at various cell cycle phases. (e-f) The sphericity of whole cell (e) and nucleus at various cell cycle phases. The cell cycle phases are indicated as shaded region in red (G1), yellow (Early S) and green (S/G2). The numbers of cells measured are N = 1,565, 437, and 924 for control, TSA, and ANA treatments, respectively.
